# A Dynamic Traffic Light Control Algorithm to Mitigate Traffic Congestion in Metropolitan Areas

**DOI:** 10.3390/s24123987

**Published:** 2024-06-19

**Authors:** Bharathi Ramesh Kumar, Narayanan Kumaran, Jayavelu Udaya Prakash, Sachin Salunkhe, Raja Venkatesan, Ragavanantham Shanmugam, Emad S. Abouel Nasr

**Affiliations:** 1 Department of Mathematics, Vel Tech Rangarajan Dr. Sagunthala R&D Institute of Science and Technology, Chennai 600062, Tamil Nadu, India; brameshkumar@veltech.edu.in (B.R.K.); nkumaran@veltech.edu.in (N.K.); 2 Department of Mechanical Engineering, Vel Tech Rangarajan Dr. Sagunthala R&D Institute of Science and Technology, Chennai 600062, Tamil Nadu, India; udayaprakashj@veltech.edu.in; 3 Department of Biosciences, Saveetha School of Engineering, Saveetha Institute of Medical and Technical Sciences, Chennai 602105, Tamil Nadu, India; sachinsalunkhe@gazi.edu.tr; 4 Department of Mechanical Engineering, Faculty of Engineering, Gazi University, 06560 Ankara, Turkey; 5 School of Chemical Engineering, Yeungnam University, 280 Daehak-Ro, Gyeongsan 38541, Republic of Korea; 6 Department of Mechanical Engineering, Fairmont State University, Fairmont, WV 26554, USA; ragavanantham.shanmugam@fairmontstate.edu; 7 Department of Industrial Engineering, College of Engineering, King Saud University, P.O. Box 800, Riyadh 11421, Saudi Arabia; eabdelghany@ksu.edu.sa

**Keywords:** multi-queuing system, signal distribution, real-time traffic scenario, convolutional neural network (CNN), traffic flow rate

## Abstract

This paper proposes a convolutional neural network (CNN) model of the signal distribution control algorithm (SDCA) to maximize the dynamic vehicular traffic signal flow for each junction phase. The aim of the proposed algorithm is to determine the reward value and new state. It deconstructs the routing components of the current multi-directional queuing system (MDQS) architecture to identify optimal policies for every traffic scenario. Initially, the state value is divided into a function value and a parameter value. Combining these two scenarios updates the resulting optimized state value. Ultimately, an analogous criterion is developed for the current dataset. Next, the error or loss value for the present scenario is computed. Furthermore, utilizing the Deep Q-learning methodology with a quad agent enhances previous study discoveries. The recommended method outperforms all other traditional approaches in effectively optimizing traffic signal timing.

## 1. Introduction

In response to the demands of a challenging society, there is increasing pressure to expand urban transportation in modern cities. As travel demand rises, traffic bottlenecks and vehicular accidents are becoming more serious. Due to unreasonable traffic signal settings, one of the most significant problems is traffic congestion. This issue lengthens passenger travel times and dramatically raises both local and global pollution levels. In order to meet the demands of challenging societies, there is pressure on the expansion of urban transportation in modern cities. As a result, one of the main problems in cities is traffic congestion, especially around signalized junctions. The cyclic model of traffic signal lights at connection junction regions uses the current traffic signal flow system, which is one of the primary causes of the problems. Even density-based traffic signal timing has been recently implemented in most modern cities. Particularly, in different parts of India, nearly 400 traffic signals have shifted to density-based traffic signal timing methods, such as: Delhi, Noida, Bhopal, Ghaziabad, Agra, Gwalior, Bulandshar, Murdabad, and Utta Pradesh (state) at Lucknow, using a hybrid method that uses an inventive algorithmic combination to determine the best times for traffic signals and can still improve obstacle avoidance. Regarding signal time optimization, traffic light control may be significantly impacted by the machine learning subfield of reinforcement learning. An approach is developed through RL to maximize rewards in a particular situation. Supervised learning consists of three main components: actions, incentives, and observations. Through the reward, an agent can communicate with its surroundings and learn whether a prior response was successful. The agent produces an image of the environment’s condition using the information it collects from the surroundings. This study presents the signal distribution control methodology for the multi-agent deep Q-network technology. Reinforcement learning is useful in creating traffic signal light control models because it can adapt field conditions based on an optimal policy. The double deep Q-learning network (DDQN) was chosen due to its ability to create traffic light control models and its recognition in several domains. Several academics have expressed different views regarding the multi-agent Q-network and the previously explored issues. Ref. [1] developed traffic signal system performance with the help of target networks and DDQN proposed, employing deep Q-network technology to regulate traffic light cycles. The signal optimization model is presented by [2] using the multi-agent reinforcement technique. A large-scale signal light system using a bootstrapped deep Q-network is shown in [3]. To illustrate the efficacy of the MDQN structure, this model produced an artificial traffic grid consisting of five-by-five crossings. In order to achieve the incentive function and efficient traffic flow of emergency vehicles in urban road network systems, a mixed reward function model for the dynamic density method was presented in [4]. A DDQN agent was used to construct the intersection cooperative traffic signal control model [5]. This model modifies the sequence in which traffic is distributed at the intersection and calculates the length of the vehicle line and total waiting time.Ref. [6] illustrated how to apply multi-agent DQNs to a knowledge-sharing deep deterministic policy gradient model (DDPG) to attain optimal signal flow. To create a multi-agent model, six distinct reinforcement learning techniques were applied to construct the experimental outcomes [7]. In [8], recommended use for the deep deterministic policy gradient (DDPG) technique relies on how large an input space deep learning system can handle. The new reward value is computed. As stated in [9], the intersecting junction is constructed as an agent structure based on a cooperative game.Ref. [10] addressed the traffic signal light controlling problem and provided innovative, rewarding solutions for a reliable traffic signal system. From a survey of the literature mentioned above, researchers mention two primary issues, namely, vehicle backlog and fixed signal timing. Particularly, [11] considered the traditional signal time, and the traditional DQN-based intelligent traffic light control (ITLC) method was used to determine the new incentive amount. At times of high demand, the current fixed signal timing flow model cannot adequately control traffic flow. Ref. [12] introduced the signal distribution control model in a dynamic vehicular system and modified signal duration based on the average number of vehicles arriving at an intersection from different directions. If the chosen direction’s arrival time exceeds the specified signal duration, the remaining time is allocated to the next direction. Thus, this paper considers dynamic traffic signal durations to compute the new incentive value. The main objective of this paper is to determine the signal timing for each phase. Traditional traffic controllers have evolved into intelligent controllers that can process and apply algorithms based on incoming traffic, delivering optimal timing patterns instead of merely acting as counters. Therefore, the aim is to analyze dynamic traffic vehicle system performance indicators for traffic management strategies in single- and multi-agent environments with low and high-traffic scenarios, such as (i) average length of the queue, (ii) cumulative vehicle waiting time (seconds), and (iii) duration of the red signal light. The remainder of the article is divided into the following sections. The simulator of the urban traffic model is covered in Section 2. Using queuing in traffic model applications (fixed-time techniques and city flow) is covered in Section 3. The proposed agent-based architecture for the problem of traffic time optimization is found in Section 4. The numerical example is discussed in Section 4, and the Results and Discussion are discussed in Section 5.

## 2. Simulator of Urban Mobility

The German Aerospace Center (DLR) created the open-source traffic simulation program known as SUMO in 2001. SUMO (Simulation of Urban Mobility) is a leading transportation simulation software known for its extensive features. It accurately models complex urban traffic scenarios and scales effectively. As an open-source tool, SUMO encourages collaborative development and customization, making it invaluable for researchers and practitioners. It has completely evolved into a tool that can be used to model future traffic by various input sources. There are two reasons the work is being released under an open-source license. While there are few available open-source traffic simulators, most were created as a part of learners and no longer maintained. In addition, there is a need to start from scratch; a significant drawback is the practically nonexistent comparability of the developed models or algorithms. Another motive for making the simulation open source is attracting other organizations’ attention. A program suite called SUMO helps with traffic simulation planning and execution. Since the traffic simulation needs to model both in their format, traffic demand representation must be produced from many sources. They are called “intelligent” because their development is predicated on skills commonly linked to intelligence, including memory, quad behavior, information processing, sensory processing, and communication. Before making changes to the infrastructure or policies, traffic simulations facilitate the evaluation process. SUMO has many extra features to aid in tasks such as mapping out routes, displaying data, importing networks, and computing emissions. SUMO allows the addition of traditional models and provides various APIs for controlling simulation remotely. The implications of autonomous route selection on the entire network are being examined in the investigation of vehicle route selection. Moreover, SUMO has been utilized to enable simulated in-vehicle telephony and evaluate the efficacy of GSM-based traffic monitoring. The V2X community heavily uses SUMO to provide precise vehicle traces and test applications in a real-time loop via a network simulator Table 1 as follows. 

## 3. Utilizing Queuing in Traffic Model Applications

The traditional mathematical study of standing in lines or queues finds its optimum use in the traffic signal timing problem. Several existing approaches have been used to thoroughly evaluate the application of queuing theory in a typical traffic scenario, and the results of these analyses have provided us with enough knowledge to create the most effective solution. Nothing more than a delay in receiving a service based on the arrival of entities to use the service can be said about the classic waiting queue.

### 3.1. Fixed-Time Technique—Traditional Traffic Management System

Traditional traffic control refers to any physical activity intended to change traffic flow on a network. Conventional traffic management alters how available road space is used to encourage safe and effective traffic flow because it may cause vehicles to cross lanes more slowly. The idea is to design a traffic management system that strikes a compromise between the increased safety and effectiveness of certain maneuvers and the delays brought on by their limits. The three categories of traditional traffic control scheme restrictions are as follows: (i) Parking restrictions (which may include stopping or waiting limits), (ii) Route restrictions (like one-way systems), and (iii) Right-of-way restrictions (like priority regulations or signals). The controller’s database contains details about signal timing techniques. Although both require wired or wireless connections, operation, and operator-selected signal timing methods, are provided by traffic-adjusted control and interconnected control. Daytime operation and operator-selected signal timing schemes are likewise possible with traffic-adjusted control and linked control, even though connections are needed via landline or cellular technology. The neighborhood intersection controller maintains a file with the traffic management center’s time-of-day control schedules. The local intersection controller extracts the system detector data, which then relays the population parameters and size to higher levels at intervals of up to one minute. Based on data from nearby sensors, the local controller finishes actuated phases at the appropriate time. This refers to the traditional three-level traffic management system used by the Department of Transportation’s FHWA Operations.

### 3.2. Deep Q-Network

**Figure 1:**
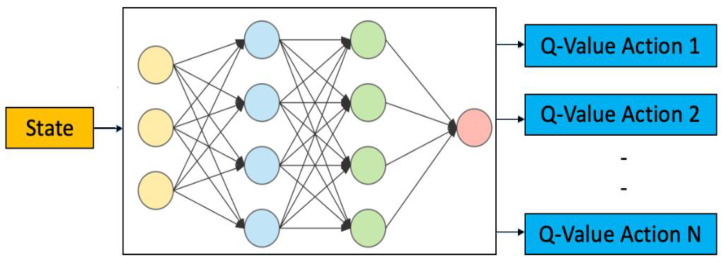
State and action framework of DQN.

In the DQN paradigm, an agent must remain on the path of its present state (t) in the given situation upon deployment. The environment is modified by [26,27,28,29] from its state when the agent uses a Markov decision process to weigh various options. When agents change based on a previous action, they are rewarded (t + 1). The agent’s goal is to select the action to maximize benefit in the given scenario. The Q-value table is maintained by typical deep Q-learning, and the entries are modified as the agent gains knowledge. The following graph displays the optimal Q*(state, agent) function. Maintaining an arrangement becomes impossible as the state space grows. Figure 1 shows the DQN architecture.

Determine the Q* function value by using DQN$\left(s,\langle a\rangle \right)$ as follows:

(1) $${Q^{*}}_{\left(s,\langle a\rangle \right)}=\;\delta *\;Inf\;Q{\;}_{\left(s+1,\;\langle a+1\rangle \right)}\;+\;r_{\left(t+1\right)}$$

The preceding action element alone can affect an action’s outcome if the system meets the requirements of the Markov process. The following equation suggests this:

(2) $$P(s+1\;||\;((s,s-1),\;\langle a,a-1\rangle ....))=(s+1\;||\;(s,\langle a\rangle ;r))$$

In general, immediate benefits will occur before long-term, delayed benefits. The agency wants to optimize total return. The following formula can be used to determine the total yield or anticipated discounted cumulative reward over time.

(3) $$r_{t}=Exp\;\left[\sum_{n=0}^{\infty }\frac{\partial^{n}(t+n)}{\partial t^{n}}\right]$$

The discount factor ∂∈[0,1] is represented by ∂.

Action state space function values are two more value functions expressed as V(S). It represents expected outcomes when implementing policies:

(4) $$v_{s}=\;Exp\;\left[r_{t}+\frac{\partial \;v_{\left(t_{i}\right)}}{\partial t}\right]$$

Estimate the Q-value of this system, in which the vector coefficients can approximate involved and restrained functions. Neural networks, “deep neural network models”, contain numerous hidden layers. Here is a diagram that shows the equation for the deep Q-network.

(5) $$Q(t+1)(s_{t},\;\langle a_{t_{i}}\rangle )=\;\Delta t+r_{i}*Q(t)(s_{t},\;\langle a_{t_{i}}\rangle )$$

(6) $$\Delta (t)=\left[\frac{\partial \;\left(Q(t)\left(s_{\left(t+1\right)},\langle a_{t}\rangle \right)\right)}{\partial t}-\frac{\partial \left(Q(t)\left(s_{t},\langle a_{t}\rangle \right)\right)}{\partial t}\right]$$

Radiant descent and backpropagation techniques periodically upgrade the neural net weights to approximate the Q-functions. It is best to reduce the amount of this mistake function (Chantal Schneider). The temporal difference aim of the formula can be used to estimate the accurate Q-value, as it is unknown. It shows the total anticipated benefit for every subsequent time step while accounting for future incentives that have been discounted. The goal value can be updated repeatedly. As seen below, the squared loss function is used to build the deep Q-network’s failure (F) function.

(7) $$F\;=\;\frac{1}{2}\left[Q(t)\left(s,\langle a\rangle \right)-\frac{\partial \left(\nu \left(s_{t+1}\right)\right)}{\partial t}+r(t)\right]$$

**Figure 2:**
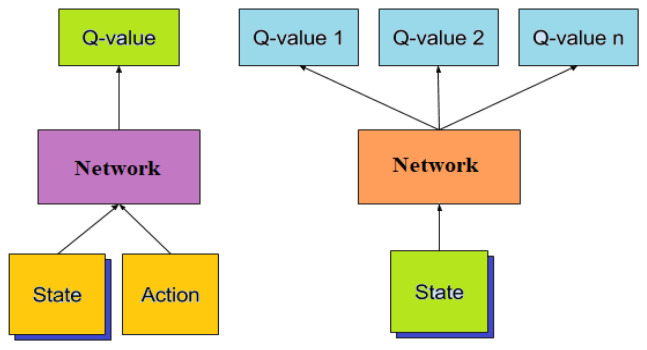
Architecture of the deep Q-network.

The deep Q-network’s anticipated value (3) it is depicted in Figure 2 and represented with the following equation:

(8) $$F_{\theta (t)}=Exp\left[\nu (t)-Q(t){\left(s,\langle a\rangle ;\theta \right)}^{2}\right]$$

This method eliminates data dependencies by randomly choosing data and storing it in the replay buffer before training is complete. However, it is not possible to store all observed data in the replay buffer at the same time. Depending on the situation, there may be an exception, but a particular experience is more valuable. The most important technique used in many kinds of research to rank or define the attributes that make learning material valuable is the prioritized experience replay. Setting up a multi-deep Q-network reinforcement learning goal that considers the agent’s ability to adapt the changing behavior of other agents and stability of its learning dynamics is difficult [30]. Several factors can influence multi-deep Q-networks’ performance. On the other hand, a reward-free setting implies a minimal likelihood of earning a unique reward confined within an odd, asymmetrical context. When rewards are given out regularly, even for unlearned conduct, there is a greater chance of success. The following equation displays the MDQNs as they are configured.

(9) $${Q^{*}}_{t}(s(t),a(t))=Q_{t}(s(t),\;a(t))+R(v),$$

where R(v)—reward value

(10) $$R(v)=\partial (r(t+1)+\gamma \;\max\;Q_{t}(s(t+1),\;a(t))-Q_{t}(s(t),\;a(t)))$$

The MDQN acquires experience faster than the standard DQN because it employs many actors to account for architectural irregularities. Learning speed is significantly impacted by using prioritized experience replay, which is based on this architecture, to assess experience value. The degree to which the current state may be wellread by comparing the objectiveinaccuracythrough the actualstatisticswith calculating the statistical metrics will determine its relevance.

(11) $$T(t)=R(t)+RT(t)-Q_{t}(s(t+1),a(t+1))$$

(12) $$RT(t)=\gamma \;\;Q_{t}(s(t),MaxQ_{t}(s(t),a(t)))$$

Before deciding on a new course of action, the agent will use a deep Q-learning technique to gather the condition of parallel crossroads. Moreover, cycle duration has been designated as (a′) for upcoming updates on the innovative datasets, contingent on data about interchange conditions at adjacent crossings. Once the new cycle length is identified and applied to change the phase signal length, the process outlined above would continue indefinitely.

### 3.3. Quad-DQN

**Figure 3:**
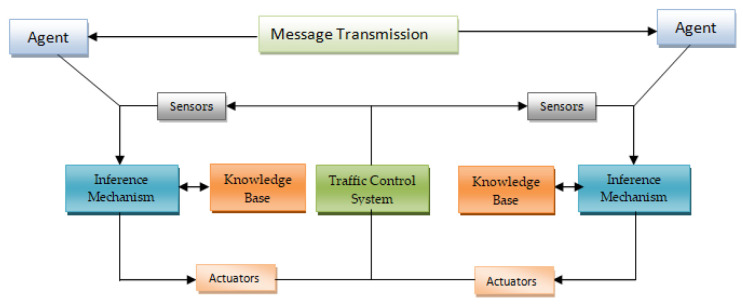
Quad agent-based architecture for a traffic control system.

Before obtaining the Q-value, the deep Q-network is split into two result values: e (function) and e′ (value). Afterwards, it is merged once more. Quad-DQN begins with two dividing components to achieve its goal. Divide the current Q-value in half first, then add the entire Q-value. The current Q-value is defined as the action value in a specific state after the agent has further broken it down. Due to the agent’s desire to maximize the future reward value and score the resource forecasting, it is interested in obtaining the future reward value from the function stream. The reward value is affected by ‘e′’ if the action value is not considered. As a result, one stream value can be determined by the state action, while the other will act on each state after learning the results. However, because the reward value for each state action varies depending on the circumstances, Q-value calculations for the general DQN method function value are not accurate. The desired result was not obtained, even though every action state operated simultaneously. Without extracting the current MDQN, the quad agent MDQS was proposed in this research. First, a comparable prerequisite was built for the current dataset. Then, the current scenario’s loss value (error) was calculated, and the value from the precondition $q_{e\;(s^{\prime })}$ alone was substituted in the MDQS. Lastly, $q_{e^{\prime }\;(s^{\prime })}$ phase selection with the lowest loss value can be performed more simply by comparing the loss value to that which was acquired [31]. To improve performance by minimizing loss, the quad Q-network compares the obtained loss value of (e, e′) with the learning rate. This model offers advantages such as enhancing the accuracy of current DQN processes and facilitating its integration into DQN algorithms. Figure 3 illustrates the new approach:

(13) $$q(s\prime )=q_{e^{\prime }(s\prime )}\left[R\left(b_{s}\right)\;,\;a(q_{e\prime (s\prime )})\right]$$

where R (b_s_) represents the batch size range, and ‘a’ represents the argument max.

(14) $$T(q(s\prime ))=R+\gamma \;*\;\max\;\left(q(s\prime )\right)$$

(15) $$L(s\prime )=\;{\left[T(q(s\prime ))\;-q{\;}_{\theta (s)}\;\;*\in \right]}^{2},$$

## 4. Quad Agent-Based Architecture for the Traffic Time Optimization Problem

**Figure 4:**
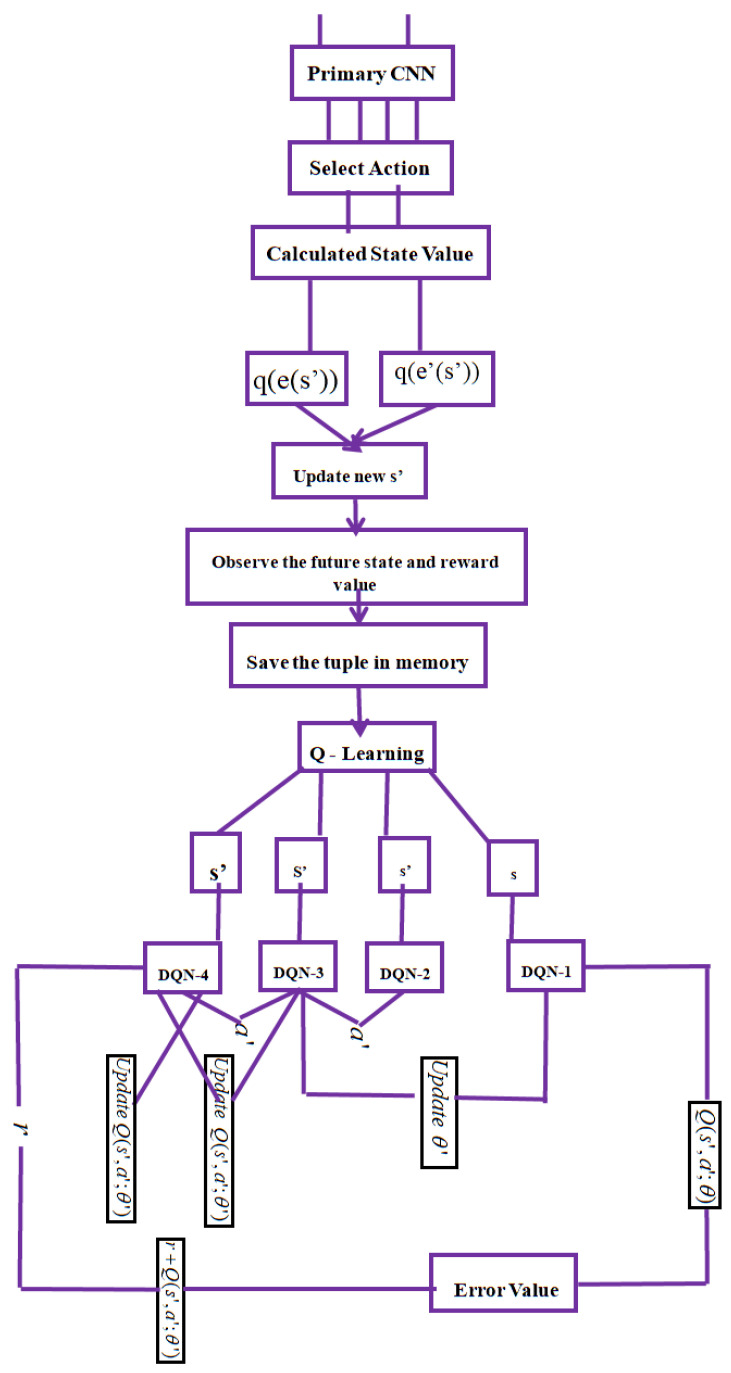
CNN for QAMDQS Flow Chart.

Multi-agent systems are valuable resources for solving issues in remote areas. The data, control functions, or both could be dispersed in a distributed system. The idea of an agent can help address scenarios where it would be challenging or even impossible for a single creature to comprehend the status of a system entirely. Several dispersed scenarios include difficulties with route load balance, traffic management, and traffic negotiation between vehicles and infrastructure. The agent-based design of the traffic control system is shown in Figure 4 below.

Although agent-based methods are increasingly common in engineering applications, their potential for sophisticated traffic control has yet to be adequately investigated. The multi-objective Markov decision process is developed and simulates agent activity. Agents can use this technique to decide on various policy goals. Several function approximation techniques have been developed using a reinforcement learning-based methodology to improve the control algorithm. A threshold ordering method is offered and integrated with the learning algorithm. Different road traffic controls may be implemented using the multi-objective intelligent control method. Different switching devices, like stoplights, ramp metering, vehicle speed, and lane correction measures, are frequently utilized in today’s traffic management on motorways and city streets. A traffic modeling framework must be used while developing a traffic management system and an agent-based modeling framework is a wise and worthwhile choice. Traffic devices can be modeled as intelligent agents interacting with the surrounding traffic in an agent-based framework. The effectiveness of the suggested model QAMDQS is evaluated by considering a range of traffic scenarios, such as sequential ideal flow traffic, continuous flow traffic on all sides, dense flow traffic on all sides, dense flow traffic just on one side, and moderate flow traffic on all sides.

### 4.1. Quad Agent Multi-Queuing System on the Signal Distribution Control Technique (QAMDQS)

This Section introduces the QAMDQS based on the signal distribution control technique. Neighbor signal phase condition and total vehicle signal flow time in the intersection region are the two parameters that are considered. The traffic jams already present at the surrounding intersections have the potential to develop because the neighboring light’s immediate next signal phase might arrive at the intersection’s junction right away. In this instance, the traffic light’s subsequent cycle length ought to be modified while considering the two different traffic signal flow circumstances. Each agent should then gather the traffic flow conditions for both the current and the next signal phase. Consequently, the proposed algorithm will run in two steps, with each agent updating the traffic signal duration length on the collected states at this time. First, each agent will use a signal distribution control algorithm to determine what to do with the gathered states. The state input of the algorithm is the current traffic condition at the intersection. If ‘s’ is selected as the action, then the traffic signal light determination time interval is applied. Finally, the current traffic situation (vehicle queue length) will be examined during the processing period. The action then modifies the duration of the traffic light at the nearest signal phase intersection junction. When the action (e,e′) is chosen, and the new state value (s′) is updated, the traffic signal light determination time interval is used. Lastly, the current traffic situation (vehicle queue length) will be examined during the processing period. The following signal phase cycle length must be implemented to achieve the concluding cycle length. The algorithm (Section 4.2) displays the framework of the recommended algorithm. The signal distribution control algorithm [12] produces an adequate signal flow by calculating the waiting time per cycle length.

### 4.2. Pseudo-Code for QAMDQSBased on the Signal Distribution Control Technique

#### Description of Proposed Algorithm

The proposed method periodically executes the signal duration when the loop condition is met. The overall cycle length and traffic vehicle waiting time from step 4 is computed using Algorithm 1. The overall system performance is typically influenced by (i) parameter values, (ii) feature datasets, (iii) error values, etc. This means that utilizing steps 5 through 14 determines the new future state value (s′) by considering the system function value (qe(s′)) and parameter value (qe′(s′)). The state value for each signal phase is gathered by the reward calculation method. The new action space (a′) is described in steps 15 through 25. The Q-value is computed using the suggested approach. Pseudo-code, state value, and action space are saved for the subsequent quadruple building starting in step 26. The parameter training process is explained in steps 27 through 29 and the state value is verified. One quadruple is created if the value is more than zero; if not, the agent receives the messages its neighbors have sent on traffic patterns at the pertinent intersection in step 30. The agents try to send a message at any moment in step 31. Step 32 determines the backlog of traffic vehicles for each phase.
**Algorithm 1.** QAMDQS based on the Signal Distribution Control Algorithm techniqueInput: Space of Action A, B Assume Parameters Output: $R_{v(t)},\;T_{t},\;RT_{t},L_{q(t)},\;w\;\;\;\;\;t\in \nu =\left\{1,2....n\right\}$ Repeat Set
1   Set: Phase = 0, Cycle = 0
2   Set T = T_Initial_
3   While True do
4   Calculate $w_{k_{i}},\;T_{c_{i}}$, $i=\left\{1,2....m\right\}$(Algorithm [13])
5   Collect the state value from the corresponding intersection cycle length
6   while
7   $T<T_{\max}$
8       Split (State(s)) = ($q_{e(s\prime )},\;q_{e\prime (s\prime )}$)//{[Function, Value]
9       $q_{s\prime }=\;q_{e\prime (s\prime )}\;[R(b_{s}),\;a(q_{e(s\prime )})]$, where b_s_- batch size, a-argmax
10       $T(q_{s\prime })=\;R\;+\;\gamma \;\max\;(q_{s\prime })$
11       $L_{q_{s\prime }}=\;[T_{q(s)}-q_{e(s\prime )}\;*\in ]$, where $\in =(\in_{\max}-\in_{\min})$
12   Update s’ from $q_{s\prime }\;\;\&\;T(q_{s\prime })$
13   If Phase > 0 then $Q_{t}(s,a;\theta )$
14       If memory exceeds the state space ‘B’, then eliminate the oldest value
15          Store the new quadruple $Q_{t}\langle s,a,\;r,s^{\prime };\theta \rangle$
16       Else
17   End if
18   Performance action $s^{\prime }$ and move to the next state and observe the reward value
19   Store this transition $Q_{t}\langle s_{i},a_{i},\;r_{i},{s_{i}}^{\prime }:\theta \rangle$ from the stored data
20       Set $r_{t}=v_{t}-v_{t+1}$
21          Set $R_{v}=\partial (r_{t+1}+\gamma \;\max\;Q_{t}(S_{t+1},\;a_{t}:\theta )-Q_{t}(S_{t},\;a_{t}:\theta ))$
22          Set $T_{t}=R_{t}+RT_{t}-Q_{t}(S_{t-1},a_{t-1}:\theta )$
23          Set $RT_{t}=\gamma Q_{t}(S_{t},\max Q_{t}(S_{t},a_{t}:\theta )$
24       Set $W=\sum_{i}^{N_{t}}w_{n}$
25   Select the action $a^{\prime }$ from A which has the maximum Q value
26   Store tuple $Q_{t}\langle s^{\prime },a^{\prime }:\theta \rangle$ for future usage
27   If Phase > 0 then
28       Update AQMDQN model based on the stored quadruples
29   End if
30   Send message to the agents that control neighbor intersection signal phase
31   Check the message received from agents that control neighbor intersection signal phase and update action space $a^{\prime }$ to obtain the final action output based on the received messages
32   direction = direction + 1
33   End while **Pseudo—Code** Function Distribute To RSUF or Signal Flow() Initialize Total Number of Vehicles to 0 Initialize Total Cycle Time to 0 While (Interval Time)  For each medium along with RSU   Increment Total Number Of Vehicles by 1   Calculate Total Cycle Time  End For End While End Function Function Collect Data By Agent Using RSU() For each data accumulation cycle  Collect uncontaminated data  Preprocess data End For End Function Function Obtain New State Value() For each accumulation cycle  Accumulate present state data value from corresponding cycle length  Set parameter values and function values  If the vehicle calculates a value greater than zero   Obtain the new state value  If memory exceeds agent capacity   Eliminate oldest data  Store the new state value for the nearest agent End For End Function Function Announce Signal Light Status (Phase, vehicle Length, period) For each phase up to Phase + 1  For each time interval up to time + 1   Set Signal Period   Set Status to ‘on’   Inform decision agent unit of (Phase, Status, period)  End For End For End Function Function Decision By Agent (Phase, period) For each phase up to Phase + 1  Set Status Of Traffic Signal to updated status End For End Function Function Evaluate System Performance (new State, reward Value) Calculate difference between two consecutive waiting times Set New State Value Set Reward Value Set Target Value Set Reward Target Value Set Total Signal Flow Time Select action agent from A with the highest Q value If Cycle Time is greater than 0  Update QAMDQS model based on stored data End If Send message to agents nearest to the intersection signal phase Check message received from agent control phase and action phase Increment Phase by 1 End Function

The Figure 4 Methodology Procedure is as follows:

➢ Quad-DQN begins with two dividing components in order to achieve its goal.
➢ Divide the current Q-value in half first, then add the entire Q-value.
➢ Due to the agent’s desire to maximize the future reward value and predicting the score value, it is interesting to find the future reward value from the function stream.
➢ The reward value affected ‘s’ by ‘e’ if the action value is not taken into account.
➢ However, the reward value for each action states varies depending on the circumstances; Q-value calculations for the general DQN method function value are not accurate and did not receive the preferred result, even though every action state was operating simultaneously.
➢ Without extracting the current MDQN, the adaptive quad agent MDQN was proposed in this research.
➢ First, a comparable prerequisite was built for the current dataset. Then, the loss value (error) of the current scenario was calculated, and the value from the precondition $q_{e\;(s^{\prime })}$ alone was substituted in the MDQN.
➢ Lastly, by comparing the loss value to that which was acquired $q_{e^{\prime }\;(s^{\prime })}$, phase selection with the lowest loss value can be performed more simply.
➢ In order to improve performance by reducing the error value and quad Q-network compares the obtained error value of (e,e′) with the learning.

Determining the Q-value for each state space is essential to this methodology. The action’s most significant Q-value will determine which action is chosen. An action space considers each action and how it relates to other actions. The proposed model considers the four phases of the state activity of the traffic signal system. Phase 1, sometimes called t1, manages the green light for north–south traffic. The green light for traveling north to south is controlled by phase 2, referred to as t2. Phase three, often known as t3, controls the green light for vehicle flows from east to west. Phase 4, sometimes known as t4, controls the green light for east-to-west movement. Changing all of the signal flow values for every phase is the main objective of this action; specifically, a select agent modifies only a subset of the values for each phase. The reward can be used to gauge how much action is adopted in reaction to modifications in the outside world because thesignal distribution control algorithm (SDCA) tackles the issue of traffic congestion; changes in traffic circumstances must be factored into the incentive value.

The length of the lines formed by backed-up vehicles, the total time instant in which the exchange has been waiting, and red signal progress, which is crucial during the entire procedure, are the three main objectives of the incentive computations—here, the focus is on queue length and the total arrival time of all the recently arriving vehicles. (i) A long waiting queue forms at intersections due to a high volume of vehicles arriving as quickly as feasible. (ii) If one only considers wait length, one will see that a busy land region exists, whereas vehicles with heavy traffic have shorter queues. This will result in a shorter phase length. In this case, a unique calculation technique that takes the queue’s length into account along with the waiting period as follows:

(16) z(t)=c(t+1)−c(t)

The two consecutive cyclic periods are represented by c(t+1)   and c(t)   and the difference between the two cyclic periods is calculated in z (t)  . The resultant value reflects the potential direction of the vehicle flow.

The total cumulative vehicle waiting time

(17) $$W=\sum_{i}^{n_{t}}w_{k_{i}}$$

### 4.3. Multi-Agent System

In the proposed QAMDQS system, the four-number type of multi-agent system is utilized. The minimization objective function is induced to the solution of linear sequences and is represented by the following equation:

(18) $$Min\;\;(\omega )=\rho -x-\sum_{\alpha =1}^{n}\omega_{\alpha }$$

where $\omega_{\alpha }(x)$ is a linear sequence and $\omega^{-1}=a+bx^{2}+\in$, where $\omega^{-1}$ is the least-squares method. Agents will select once from random and linear regression for service. The ‘x’ is the waiting time of the individual vehicle. V is the waiting time distribution using the factor of the cumulative distribution:

$$A(U)=AV,\;\;V\in \left[0,\;U_{1}\right]$$

where $U_{1}$ is the estimated waiting time distribution.

### 4.4. Performance Measures for Traffic Management Techniques

The following equation calculates the QAMDQS system’s performance using the average number of vehicles entering all lanes, the average number of vehicles entering a given lane, the average waiting queue in all lanes, and the average waiting time in that lane.

➢ The average number of vehicles entering in all lanes is
  (19) $$\rho +\left(\rho^{2}/2(1-\rho )\right).$$
  λ is the arrival rate, μ is the service rate, and ρ is the utilization time.
➢ The average number of vehicle entries in the specific lane is
  (20) $$\left(\rho^{2}/2(1-\rho )\right).$$
➢ The average waiting line in all lanes is
  (21) $$\left(1/M\right)+\left(\rho /24(1-\rho )\right).$$
➢ The average waiting time in the specific lane is
  (22) $$\left(\rho /2M(1-\rho )\right).$$

The total amount of time, in seconds, spent waiting for red and orange lights when a vehicle is snarled in traffic is known as the cumulative waiting time, defined as:

(23) $$C\left(WT_{i}\right)=RT_{i}+OT_{i}$$

where $RT_{i}$ represents the red signal time of ‘*i*th’ duration, and $OT_{i}$ represents the orange signal time of ‘*i*th’ duration.

➢ Average waiting time: the average of the entire vehicle’s waiting time in seconds in traffic is the average waiting time:
  (24) $$E\left(WT_{n}\right)=\sum_{i=1}^{n}C\left(WT_{i}\right).$$

A red signal is typically used to indicate threat or caution, a traffic light that indicates a potential danger ahead by serving as a signal for drivers to stop. The amount of time that the red signal is in an active state is referred to as the duration of red signal light.

## 5. Numerical Example

The experimental setup was constructed using SUMO, and a standard piece of software for simulating traffic settings that closely mimic actual situations is called SUMO. Three lanes are used in this model’s four perpendicular street scenario for commuter traffic. Each has three lanes: the leftmost is for left turns, the rightmost is fixed for right turns, and the middle is for vehicles going ahead. With the lengths set at 100 m, an intersection area measuring 200 by 200 m was considered. They should be separated by at least two meters, and the vehicle should be five meters long. The 40 × 40 grids are five meters in size. Every vehicle arrives randomly, with a predefined arrival time of one-tenth of a second. Traffic moving in all directions (west, east, north, and south) will proceed at a pace of two-tenths of a second because two through-pass lanes are available. The vehicle has a maximum speed of 30 km/h across the intersection, and its acceleration and deceleration rates are 1.0 and 4.5 m/s^2^, respectively. The experimental setup hyper parameters are mentioned in Table 2. The proposed technique is evaluated using data from the Solinganur signal station in Tamil Nadu, India. The public can access the dataset since it meets the requirements of the open data policy. The daily traffic volumes in Solinganur are measured every ten minutes. The sensors’ placement and the flow direction are two further crucial factors. The following variables are meticulously gathered and examined: the time, day order, intersection phase, quantity of passing vehicles, and cycle time.

**Figure 5:**
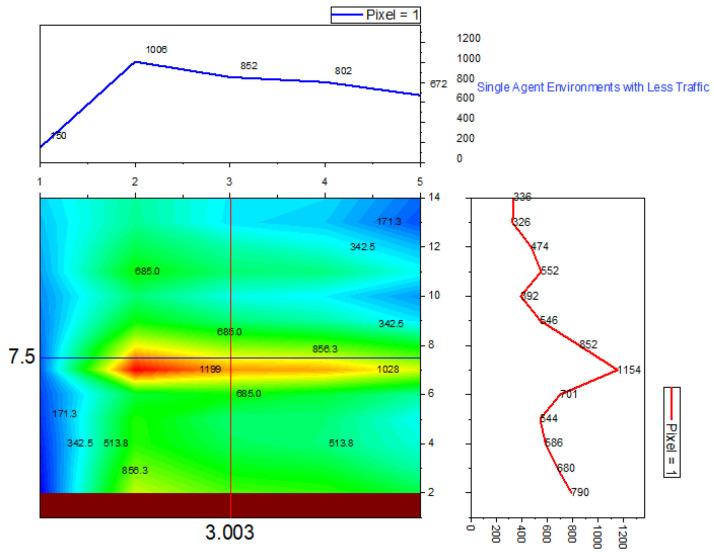
Single agent less traffic.

**Figure 6:**
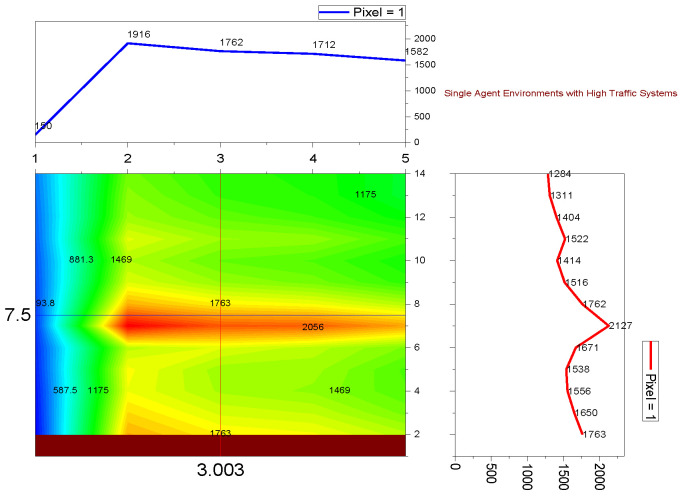
Single agent heavy traffic.

**Figure 7:**
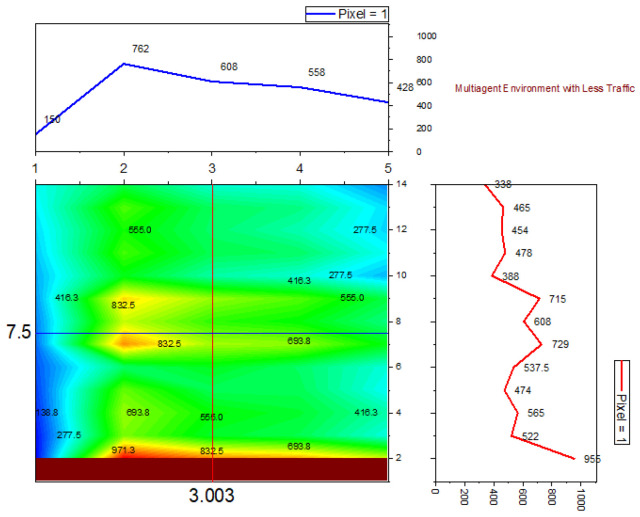
Multi-agent less traffic.

**Figure 8:**
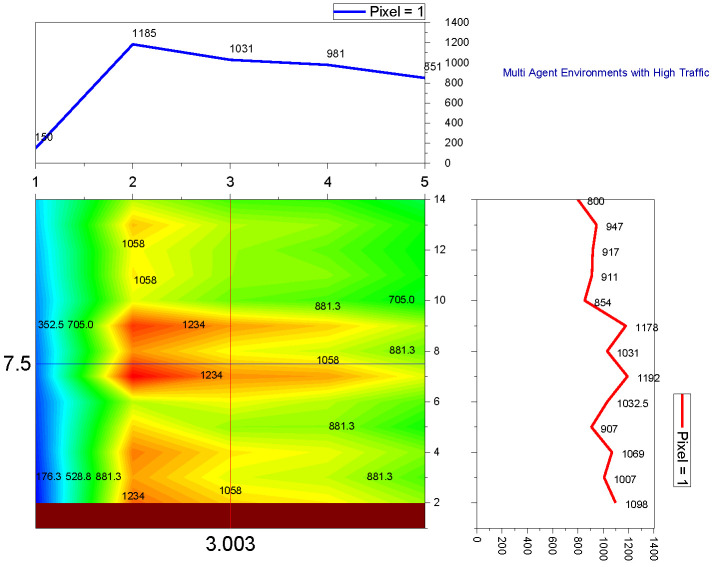
Multi-agent—heavy traffic.

➢ Here, traffic signal cycle time and service rate are related.
➢ The proposed model evaluates the effectiveness of the traffic signal system. DQN, cityflow, and fixed time are used in a single-agent setting with modest traffic volume, which outperforms the proposed method.
➢ In comparison to other contemporary systems, the QAMDQS has a minimum waiting time of 156 s, as seen in Figure 5. Compared to other existing systems, the queue waiting time in a situation with a single agent in high traffic is 1104 s, as shown in Figure 6.
➢ Similarly, in the multi-agent system the waiting time is 158 s, inadequate when compared to the other techniques. In the multi-agent environment with significant traffic, the proposed method provides an impressively short waiting time of 620 s.
➢ Performance analysis is conducted at different stages. The suggested QAMDQS approach is applied in a simulated traffic environment with single and multiple agents. The multi-agent queue system operates better than the single-agent queue system, as illustrated in Figure 7 and Figure 8.
➢ The suggested QAMDQS model outperforms the fixed-time method, CityFlow, and DQN in terms of performance metrics, with cumulative vehicle waiting time, average queue length, and red signal light duration measuring 53, 84, and 59 s, respectively.

Based on the experimental setup, the queuing system’s waiting time is significantly reduced to 620 s when applied in a high-traffic multi-agent environment, compared to existing methods. Performance analysis involves assessing both single-agent and multi-agent queuing systems. In real-time traffic scenarios, the multi-agent queuing system exhibits a minimal cumulative vehicle waiting time of 368 s, surpassing the performance of the single-agent queuing system method.

**Figure 9:**
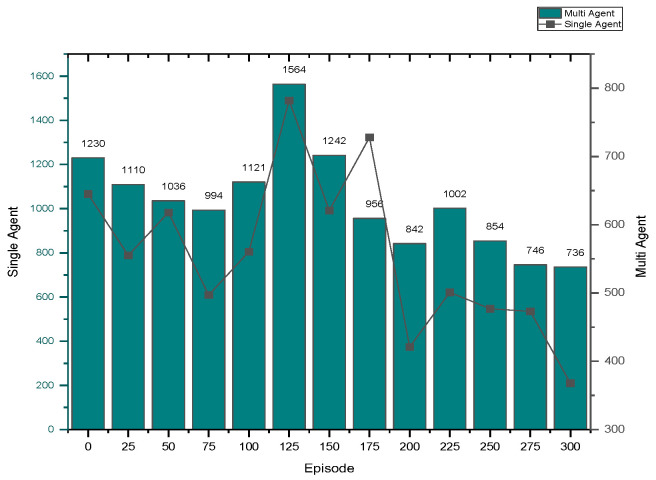
Single-agent vs. multi-agent.

Utilizing the heat wave transition of single- and multiple-agent functions, the system function analyzer determines queue waiting times in red and maximizes the flow of arriving vehicles in green. In Figure 5, Figure 6, Figure 7 and Figure 8, blue represents moderate flow, red denotes light and heavy traffic scenarios, and green indicates the highest service flow. Figure 9 compares real-time traffic situations for single- and multi-agent systems. Using the normal distribution, the maximum probability of green signal flow for multi-agent systems is obtained. This signifies that QAMDQS efficiently communicates signal transitions to the nearest signal station. Therefore, the suggested technique significantly enhances the performance of the multi-agent queuing system. Thanks to the advantages of the quad multi-deep Q-network model, signals for the next closest station are communicated swiftly.

**Figure 10:**
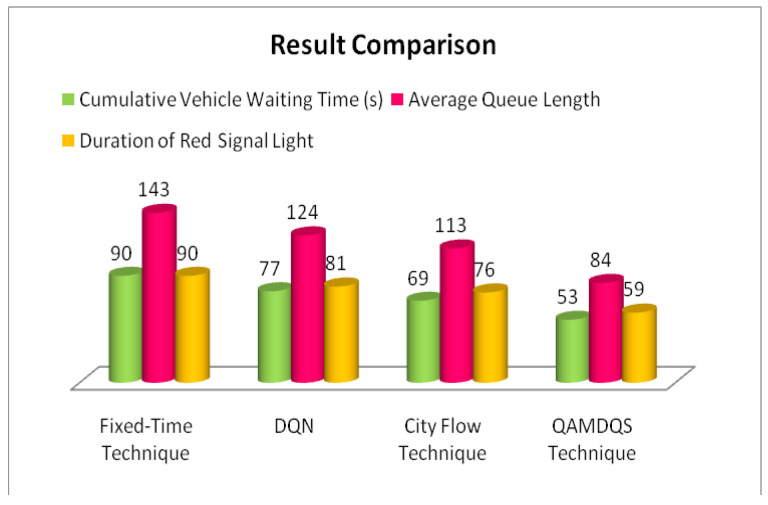
Result comparison.

The system’s performance is tested using various techniques: (i) the fixed-time technique, (ii) DQN, (iii) city flow techniques, and (iv) the QAMDQS technique. When applying the simulated traffic environment to the proposed QAMDQS technique with single and multiple agents, performance evaluation is conducted at different time intervals of the episode. It is observed that the multi-agent queue system outperforms the single-agent queue system. Table 3 QAMDQS method is assessed for cumulative vehicle waiting time, average queue length, and duration of red signal lights in the simulated traffic environment. The results show 53 s, 84 s, and 59 s, respectively, as depicted in Figure 10.

## 6. Discussion of Research Results

The paper’s focus is on designing and implementing innovative traffic signal timing algorithms along with operational strategies to handle growing global traffic volumes. Given population growth and a prevailing preference for personal transportation over public transit, optimizing traffic signal timings poses a significant challenge. The paper aims to develop a methodology that integrates reinforcement learning with traffic signal light time optimization. Real-time traffic data in simulation scenarios can be captured using object detection techniques with cameras integrated into traffic light controllers. The paper assumes that the green signal state represents an open queue state, assuming no waiting time for vehicles. The contribution lies in proposing the quad agent multi-queuing system on the signal distribution control technique (QAMDQS), a hybrid of conventional queuing techniques, integrating multiple agents with a deep Q-network for learning. The model’s adaptiveness is showcased through its integration of a multi-agent approach for cooperative processing within the queuing system. The novelty of the approach lies in deploying four agents on a four-junction road, with each agent controlling the redlight duration in different directions. These agents learn patterns in real-time dynamic vehicle movement using the SUMO simulator. Real-time traffic simulation using SUMO is employed to implement QAMDQS, utilizing 200 iterations to create a realistic simulation setup. Time is segmented into episodes, each spanning one hour. Following the implementation of QAMDQS, performance metrics including cumulative vehicle waiting time, average queue length, and duration of red signal lights demonstrate reduced waiting times compared to fixed-time, deep reinforcement, and city flow methods. This underscores QAMDQS’s effectiveness in minimizing waiting times at traffic signals. The model showcases efficiency without necessitating additional setup in existing traffic conditions, thereby promoting economic feasibility in the market.

## 7. Conclusions

This paper focuses on developing and implementing innovative traffic signal timing algorithms and operational methods for traffic signal controllers. A significant challenge for researchers is optimizing traffic signal timings, exacerbated by population growth in major economies and a preference for personal mobility over public transit networks. The paper integrates reinforcement learning with traffic signal light optimization. Cameras integrated into traffic signal controllers use object detection techniques to capture real-time traffic in simulation scenarios. The green signal condition is assumed to represent an open queue state, in contrast to the red signal condition where vehicles are identified as waiting. Performance analysis involved using both single-agent and multi-agent queuing system environments to evaluate results. Compared to the single-agent queuing system technique, the multi-agent queuing system demonstrated significantly shorter cumulative vehicle waiting times, at just 368 s, when applied to real-time traffic scenarios over multiple hour-long episodes. Compared to the fixed-time method, deep reinforcement method, and City Flow method, the proposed QAMDQS model consistently showed improved performance metrics for cumulative vehicle waiting time (53 s), average queue length (84 s), and duration of the red signal light (59 s). While the QAMDQS models performed exceptionally well, accommodating various traffic combinations, including two- and three-wheelers, remains a challenge that future research should address. To overcome the limitations of testing algorithms solely in simulated environments, the developed method will undergo further evaluation in real-time, complex network scenarios.

## Figures and Tables

**Table 1:** Summarizes researchers undergoing traffic signal timing optimization using the queuing theory model.

Literature	Methodology	Simulator	Objective
Alam et al. (2021) [13]	Open Jackson queuing model	SUMO	Waiting period, arrival time, and utilization rate
Bi et al. (2021) [14]	Unified queuing and neural network model	SUMO	Reward system to determine the most time- and energy-efficient courses of action
Blazek et al. (2021) [15]	Analytical semi-Markov queuing model	SUMO	Stochastic arrival times and a finite service time
Oke et al. (2020) [16]	Queuing intersection algorithm	SUMO	Optimizing arrival rate, service rate, traffic intensity, and the mean time
Films et al. (2019) [17]	Markovian queuing model	SUMO	Calculate the travel times during rush hour, the average travel times, and savings in peak time signal timings
Motie et al. (2018) [18]	Queuing framework for horizontal traffic by the Kullback–Leibler (K–L) algorithm	SUMO	Analyzing the inter-vehicular spacing, inter-departure time
Y. Wang et al. (2017) [19]	M/G/C/C state-dependent queuing model	SUMO	Maximize average long-term passenger throughput
Zhao et al. (2015) [20].	Optimal genetic algorithm	SUMO	Travel time
Leizhen Wang et al. (2023) [21]	Human-centric multimodal deep (HMD) traffic signal control	SUMO	Minimizing vehicle delay and queue length,
Mohammad Noaeen (2022) [22]	Reinforcement learning in urban network traffic signal control	SUMO	providing statistical and conceptual knowledge based on qualitative and descriptive data analysis
AzzedineBoukerche et al. (2021) [23]	Max pressure-based cooperative traffic signal control method	Review, not specified	Addressing the data transmission delay issue by decreasing the discrepancy between the real-time and delayed traffic conditions
Ammar Haydari et al. (2020) [24]	Deep reinforcement learning for intelligenttransportation systems	Not specified	Controlling transportation systems through reinforcement learning (RL) approaches is gaining popularity in both industry and academia
Jincheol Lee et al. (2019) [25]	Joint control of traffic signals in a transportation network	TSC and RL	Measure the travel time

**Table 2:** Hyper-Parameter List used in the MMDQN system network.

Hyper-Parameter	Value
Δ Time step for traffic simulation (s)	10
Δt Time interval for RL algorithm (s)	30
T initial warming-up period for each episode (s)	450
Update rate objective (s)	100
Ratio of objective updates	0.001
Actor acquisition cost	0.0001
Quickness of critic acquisition	0.001
Memory buffer size	100,000
Minibatch volume	64
Quantity of the rebate γ	0.99

**Table 3:** Efficiency comparative analyses of existing and proposed models.

Technique	Cumulative Vehicle Waiting Time (s)	Average Queue Length	Duration of Red Signal Light
Fixed-Time Technique	90	143	90
DQN	77	124	81
City Flow Technique	69	113	76
QAMDQS Technique	53	84	59

## Data Availability

Data are contained within the article.
